# Is Surgical Treatment an Option for Locally Advanced Cervical Cancer in the Presence of Central Residual Tumor after Chemoradiotherapy?

**DOI:** 10.1055/s-0040-1701459

**Published:** 2020-01

**Authors:** Samet Topuz, Alpaslan Kaban, Seden Küçücük, Yavuz Salihoglu

**Affiliations:** 1 Department of Gynecological Oncology, Istanbul University, Fatih, Istanbul, Turkey

**Keywords:** cervical cancer, brachytherapy, chemoradiotherapy, recurrence, resistant tumor

## Abstract

**Objective** To evaluate the outcomes of surgical treatment in patients with chemoradiotherapy (CRT)-resistant and locally advanced cervical cancer (LACC).

**Methods** Patients with LACC who underwent surgery due to resistance to CRT between 2005 and 2015 were reviewed retrospectively. Disease-free survival (DFS) and overall survival (OS) related factors were analyzed.

**Results** A total of 23 patients were included in the study and the median age was 51 years old. A total of 14 patients (60.8%) experienced recurrence; among these recurrences, 8 of them were local, 5 were distant, 1 was both distant and local. A total of 9 patients (39%) died. The Median DFS and OS durations were 15 and 32 months, respectively. A total of 17 patients (74%) had undergone simple hysterectomy, 4 (17%) radical hysterectomy, and 2 (9%) total pelvic exenteration. Postoperative grade 3 and 4 complications were seen in 12 patients (52%). Macroscopic tumor presence in the pathology specimen was associated with distant recurrence and positive surgical margins with local recurrence (Log-Rank test *p* = 0.029 and *p* = 0.048, respectively). The only factor associated with OS was surgical margin positivity (Log-Rank test *p* = 0.008). The type of surgery, grades 3 and 4 postoperative complications, brachytherapy, and tumor histology were not associated with recurrence.

**Conclusion** In patients with LACC, hysterectomy is an option in the presence of a central residual tumor after CRT. However, the risk of grades 3 and 4 complications of performed surgery is high. The presence of macroscopic tumor in the pathology specimen and positive surgical margins are poor prognostic factors. The goal of the surgeon should be to achieve a negative surgical margin. It does not seem important if the surgery is simple or radical.

## Introduction

The incidence of invasive cervical cancer is declining, especially in developed countries, due to successful screening programs and HPV vaccination. Introduction of chemoradiotherapy instead of radiotherapy alone ~ 20 years ago, which was shown to be beneficial to survival, is a major advance in locally advanced cervical cancer (LACC) therapy.^1^ However, the relative survival rate of these patients seems to have remained unchanged over the last 40 years, according to The Surveillance, Epidemiology, and End Results (SEER) Program of the National Cancer Institute data.^2^

The standard treatment approach in LACC (stage 1B2–4A) is external beam radiation therapy (EBRT) followed by intracavitary brachytherapy (ICBT) and concomitant cisplatin-based chemotherapy.^3^ ^4^ ^5^ Studies showed that a response between 80 and 90% is obtained with this treatment.^6^ ^7^ ^8^ Survival rates in these patients were reported as between 67 and 83%.^9^ ^10^ ^11^ A small group of patients with cervical cancer can be unresponsive to CRT. In these patients, a residual tumor is detected in the cervix after chemoradiotherapy (CRT) and is considered to be a CRT-resistant tumor. These patients are considered to have a poor prognosis, such as patients with recurrent cervical cancer. According to some studies, 90% of relapses occur within 3 years, and the 5-year survival rate is < 5%.^12^ ^13^ There are no strong recommendations and evidence for the optimal treatment for patients with resistance to CRT. Possible options are simple hysterectomy, radical hysterectomy, or extended surgeries such as pelvic exenteration; moreover, when surgery is not feasible, other treatment approaches are chemotherapy and reirradiation issue. Lack of sufficient knowledge due to the small patient population is a limitation on this topic. According to the Dindo et al^14^ classification, grade 3 complication has been defined as any postoperative complication requiring surgical, endoscopic or radiological intervention under general anesthesia.

In the present study, we reviewed the clinical and survival outcomes of the patients with LACC who had undergone surgery due to resistance to CRT. Clinicopathological factors such as tumor size in the pathology specimen (as macroscopic, microscopic or tumor-free), the presence of tumor in surgical margin, operation type, grade-3 complication, brachytherapy, and histology of the tumor were analyzed.

## Methods

Patients with LACC who were operated for CRT resistance at the Oncology Center of the Istanbul University between 2005 and 2015 were included in the study. Patients had received CRT for LACC. Due to lack of complete response to CRT, the patients had been referred to surgery. A total of 25 patients who had been operated with a diagnosis of residual tumor following CRT were identified. One patient was excluded from the study due to a postoperative pathological diagnosis of endometrial carcinoma and due to loss to follow-up. Demographic, clinicopathological, and follow-up data of 23 patients were recorded.

### Initial Evaluation and Chemoradiation Protocol

All of the patients had a pathological diagnosis of invasive cervical cancer with cervical biopsy. After diagnosis, the patients were classified as stage 1B2 to 4A according to the International Federation of Gynecology and Obstetrics (FIGO) staging system by gynecological examination, magnetic resonance imaging (MRI), and positron emission tomography (PET). These patients were defined as having LACC. The patients diagnosed as having LACC had been started on a general treatment protocol including pelvic EBRT, 1.8–2 Gy per fraction, total 45–50 Gy with cisplatin 40 mg/m2/week and ‘3D conformal HDR brachytherapy 5 Gy once weekly × 5 weeks to high-risk clinical target volume’ in the Department of Radiation Oncology. These patients had been evaluated with gynecological examination and MRI at the end of EBRT, before brachytherapy. In the absence of expected tumor regression in the cervix according to postCRT MRI, the CRT-resistance of tumors was accepted. The patients with clinical and/or radiological presence of residual tumor in the cervix had been defined as patients with CRT-resistant tumors and therefore had undergone surgery. The patients were offered to have surgery if the brachytherapy could not be applied or completed. The evaluation of these patients had been performed in weekly organized gynecological oncology meetings of the faculty, with the participation of medical oncology, radiation oncology, gynecologic oncology, radiology, nuclear medicine and gynecopathology teams.

### Evaluated Data

External beam radiation therapy (EBRT) doses and fractions, doses of external boost radiotherapy given to patients unable to receive brachytherapy, as well as doses and fractions of brachytherapy were recorded separately for the study.

Age, height, weight, parity, coitarche, smoking status of the patients, as well as the presence of chronic disease, were recorded. Duration between the last radiotherapy dose and surgery, type of the surgery, and postoperative grades 3 and 4 complications were also recorded.

Several data were collected from pathology reports, including presence of tumor in the specimen (macroscopic, microscopic or tumor-free), histological subtypes, and presence of tumor in surgical margins. Sites of recurrence, duration until recurrence, and recurrence treatment were recorded in patients with relapse. Recurrences were classified as local if they were detected in the pelvis, cervix, or vagina and as distant if they were detected in extrapelvic locations. Disease-free survival (DFS) and overall survival (OS) analyses were performed. The influence of the following criteria on recurrence was analyzed: 1–tumor size in the pathology specimen; 2–surgical margin; 3–operation type; 4–presence of grade 3 complication; 5–whether brachytherapy was administered or not; and 6–tumor histology. According to the Dindo et al^14^ classification, grade 3 complication has been defined as any postoperative complication requiring surgical, endoscopic or radiological intervention under general anesthesia. The association between these criteria and DFS or OS was examined using the Kaplan-Meier survival analysis. The period between the date of operation and the date of last visit or death of the patient was recorded as follow-up time. Disease-free survival was defined as the period between the time of surgery and the observation of the recurrence. Overall survival was the time between the surgery and death, and follow-up time was evaluated as the time between the surgery and the time that the patient was last examined (death or last visit).

### Statistical Analysis

IBM SPSS for Windows, Version 21 (IBM Corp., Armonk, NY, USA) was used to perform all analyses. When evaluating the study data, besides descriptive statistical methods (mean, standard deviation [SD], median, frequency, percentage, minimum, and maximum), the Fisher exact test was used to compare two groups. To assess survival, the Kaplan-Meier survival analysis was performed. A *p-*value < 0.05 was considered statistically significant. Because the present study is a retrospective review, permission of the local ethics committee was not sought. However, all of the patients signed an informed consent form that allowed our center to use their clinical data for scientific trials.

## Results

Table 1 shows age, height, weight, body mass index (BMI), parity, coitarche, smoking status, presence of chronic disease, and brachytherapy administration data. A total of 9 patients had not received brachytherapy (7 due to gross residual tumor, 2 due to closed cervical canal). Of these 9 patients, 8 had received between 10 and 20 Gray external boost radiotherapy. One patient had received neither brachytherapy nor external boost (only EBRT had been administered). As shown in Table 2, 73.9% (*n* = 17) of the patients had undergone simple hysterectomy, 17.4% (*n* = 4) radical hysterectomy, and 8.7% (*n* = 2) total exenteration. Grade 3 surgical complications had been seen in 52.1% (*n* = 12) of the patients. These included gastrointestinal system (GIS) injuries requiring colostomy / ileostomy (2 patients), infection requiring relaparotomy (2 patients), urinary tract injury requiring nephrostomy (2 patients), rectovaginal fistula (2 patients) and vesicovaginal fistula (4 patients). A total of 10 out of 12 cases of grade 3 complications occurred after hysterectomy, and 2 of them were after radical surgery. The complications rate was 10/17 for simple hysterectomy, and 2/6 for radical surgery.

**Table 1:** Characteristics of the patients

Variables		
Age (years old) Min-Max ; median Parity Min-Max ; median	31–68 0–8	51 2
Height (m) Min-Max ; mean ± SD	1.50–1.76	1.61 ± 0.07
Weight (kg) Min-Max ; mean ± SD	55–105	67.1 ± 11.2
BMI (kg/m^2^) Min- Max ; mean ± SD	20.2–41.0	25.9 ± 4.5
Smoker (n, %)	12	52.1
Coitarche (years) Min-Max ; mean ± SD	15–27	20.1 ± 3.6
Brachytherapy administered (n, %)	14	60.8
not (n, %)	9	39.2
Reason for no brachytherapy, (n)		
gross tumor in cervix	7	
closed cervical canal	2	
Brachytherapy dose (Gray/fraction)	10–25/2–5	
External boost radiotherapy, (n) yes no	8 1	
External boost radiotherapy dose (Gray)	10–20	
Time between last radiotherapy and operation min-max; median	1–12	3

**Table 2:** Clinicopathological features

Feature	*n* (%)
Operation type	
Simple hysterectomy	17 (73.9)
Radical hysterectomy	4 (17.4)
Exenteration	2 (8.7)
Surgical margin	
Positive	10 (43.5)
Negative	13 (56.5)
Grade-3 complication*	
Yes	12 (52.2)
No	11 (47.8)
Pathology specimen	
Tumor-free	5 (21.7)
Microscopic	4 (17.3)
Macroscopic	14 (60.8)
Histology	
Squamous	16 (69.5)
Other*	7 (30.5)
Recurrence	
Yes	14 (60.8)
No	9 (39.2)
Site of recurrence	
Local	8 (34.7)
Distant	5 (21.7)
Local + distant	1 (4.3)
Final status	
Dead	9 (39.1)
Living with disease	5 (21.7)
Healthy	9 (39.1)

* 5 patients with adenocarcinoma, 1 patient with glassy cell carcinoma, 1 patient with small cell carcinoma.

### The Relation between the Rate of Complication and the Period from the Last Radiotherapy until Surgery

Fewer complications were seen in patients who were operated within the first 2 months (33.3% versus 64.3%). A total of 9 patients in the first 2 months and 14 patients after 2 months were operated. A total of 3 (33.3%) out of 9 patients operated within the first 2 months and 9 (%64.3) out of 14 patients operated after 2 months had experienced grade 3 complications.

### Pathological Evaluation Results

The tumor had been detected microscopically in 17.3% (*n* = 4) of the patients and macroscopically in 60.8% (*n* = 14). The pathological evaluation had revealed no tumor in 21.7% (*n* = 5). The surgical margins had been positive in 43.4% (*n* = 10) of the patients and negative in 56.5% (*n* = 13). The histological diagnosis had been squamous in 69.5% (*n* = 16) and nonsquamous in 30.5% (*n* = 7; adenocarcinoma in 5 patients, glassy cell carcinoma in 1, and small cell carcinoma in 1) (Table 2).

### Clinical Outcomes

Relapse had occurred in 14 (60.8%) patients, 9 of whom had deceased (39.1% of all patients). No patient had died due to causes other than the disease. Five patients had been classified as living with disease (21.7% of all patients) and 9 (39.1%) as healthy (Table 2). At the time of diagnosis of recurrence, 8 patients had local, 5 had distant and 1 had both local and distant recurrence. The approach to 8 patients with local recurrence had been surgical treatment (total exenteration) in 2, supportive care in 1, and medical treatment (systemic chemotherapy) in others. One patient had refused treatment. The findings of the patients with distant recurrence are shown in Table 3. Positive surgical margins, operation type, presence of macroscopic tumor in pathology specimen, occurrence of grade 3 complication, whether brachytherapy had been administered or not, and tumor histology were analyzed in terms of relation to recurrence. The presence of macroscopic tumor in the pathology specimen was found to be related to recurrence (*p* = 0.029) (Table 4). When the factors associated only with local recurrence were investigated separately, the only factor that was found to be related to local recurrence was positive surgical margins (*p* = 0.048) (Table 5). The comparison of simple hysterectomy and radical hysterectomy in terms of micro- or macroscopic tumors, surgical margins and complications is presented in Table 6.

**Table 3:** The outcomes of 5 patients with distant recurrence

Site of recurrence	Treatment for recurrence	Final status
Lung	Chemotherapy	deceased
Adrenal gland	Adrenalectomy + chemotherapy	alive
Extensive intraabdominal implantation	Chemotherapy	deceased
Brain + local	Supportive care	deceased
Brain + lung + bone	Radiotherapy and chemotherapy	deceased
Brain + lung	Radiotherapy and chemotherapy	deceased

**Table 4:** Analysis of factors affecting distant recurrence

Variables		*p-value*	OR	95.0%CI for OR	
				Lower	Upper
Pathology specimen	Tumor-free/Microscopic Macroscopic	0.029	7.750	1.226	48.984
Surgical margin	Negative Positive	0.203	2.669	0.589	12.086
Operation type	Simple hysterectomy Radical surgery	0.067	4.991	0.894	27.857
Grade-3 complication	Yes No	0.659	1.353	0.354	5.173
Brachytherapy	Yes No	0.821	1.208	0.234	6.238
Histology	Squamous Non-squamous	0.485	1.737	0.369	8.183

Abbreviations: CI, confidence interval; OR, odds ratio.

**Table 5:** Analysis of factors affecting only local recurrence

Variables		*p-value*	OR	95.0%CI for OR	
				Lower	Upper
Pathology specimen	Tumor-free/Microscopic Macroscopic	0.297	3.350	0.345	32.571
Surgical margin	Negative Positive	0.048	15.635	1.027	237.966
Operation type	Simple hysterectomy Radical surgery	0.453	1.989	0.330	11.976
Grade-3 complication	Yes No	0.111	3.998	0.726	22.025
Brachytherapy	Yes No	0.210	5.147	0.397	66.803
Histology	Squamous Non-squamous	0.497	2.096	0.248	17.723

Abbreviations: CI, confidence interval; OR, odds ratio.

**Table 6:** Comparison of simple hysterectomy and radical hysterectomy in terms of micro- or macroscopic tumor, surgical margins, and complications

Variables		Type of surgery		
		Simple	Radical	*p-value*
Grade3 complication	No	7	4	0.371
	Yes	10	2	
Surgical margin	Negative	10	3	1.000
	Positive	7	3	
Tumor specimen	Microscopic/tumor-free	8	1	0.340
	Macroscopic	9	5	

### Survival Analysis

The median follow-up period of 23 patients was 20.0 months (6–118 months) and the median OS was 32.0 ± 8.1 months (95% confidence interval [CI]: 16.0–48.0). The 2-year OS ratio was calculated as 63.3%. A total of 14 patients (60.8%) had disease recurrence. The median DFS was 15.0 ± 4.4 months (95% CI: 6.3–23.6). The 2-year DFS rate was 29.1%.

### Disease-free Survival and Overall Survival with Regard to Surgical Margin

**Fig. 1:**
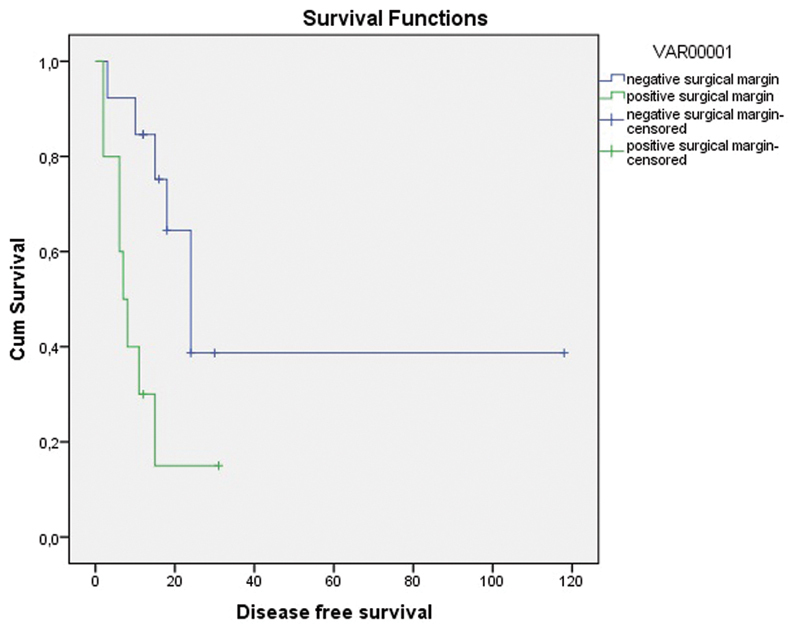
Disease-free survival graph with regard to surgical margin (months).

Recurrence had occurred in 8 (80%) out of 10 patients with positive surgical margins and in 6 (46.2%) out of 13 patients with negative surgical margins. The median DFS was 7.0 ± 1.58 (95% CI: 3.9–10.0) months with positive surgical margins and 24.0 ± 3.8 (95% CI: 16.4–31.5) with negative surgical margins (log rank test *p* = 0.048) (Fig. 1).

**Fig. 2:**
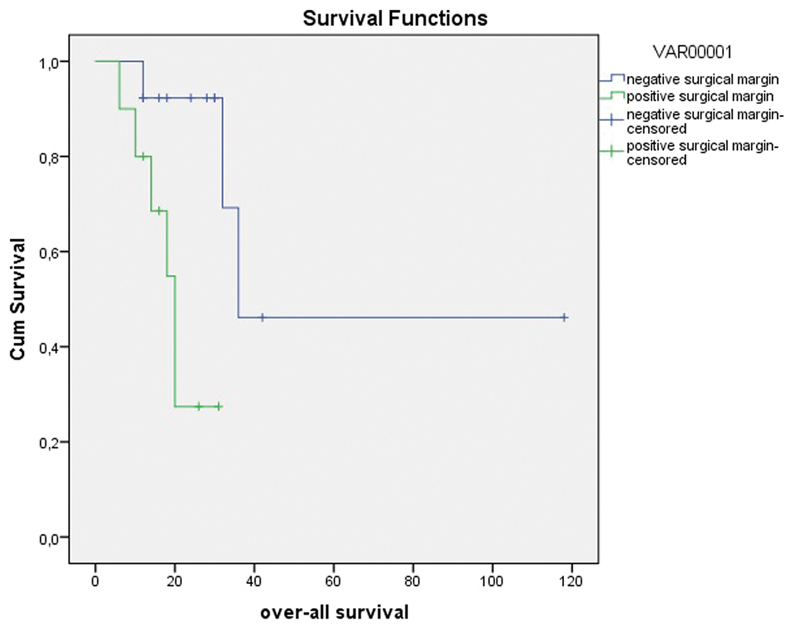
Overall survival graph with regard to surgical margin (months).

A total of 4 (40.0%) out of 10 patients with positive surgical margins and 10 (76.9%) out of 13 patients with negative surgical margins were alive. The median OS was estimated as 20.0 months with positive surgical margins and as 36.0 months with negative surgical margins (log rank test *p* = 0.008) (Fig. 2).

### Disease-free Survival and Overall Survival with Regard to the Presence of Macroscopic Tumor

**Fig. 3:**
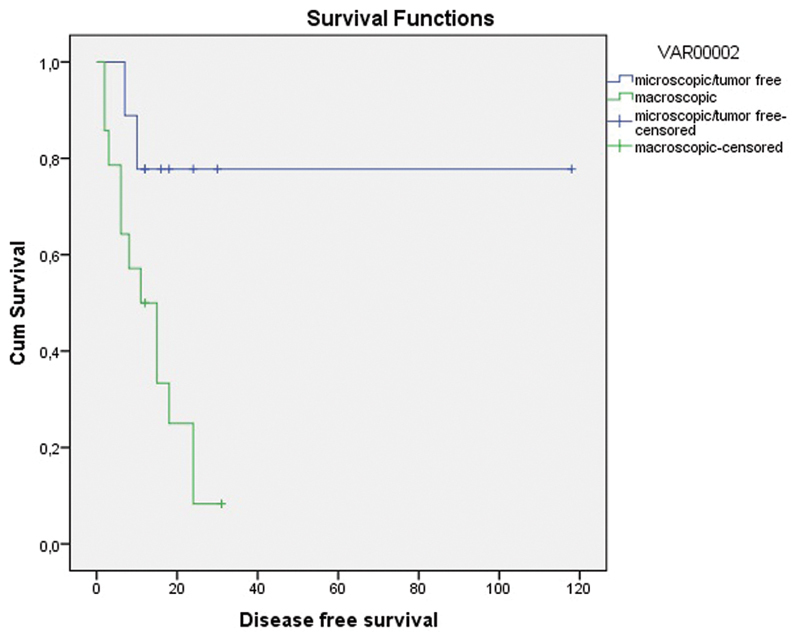
Disease-free survival graph with regard to presence of macroscopic tumor in the pathologic specimen (months).

Recurrence had occurred in 12 (85.7%) out of 14 patients with macroscopic tumor and in 2 (22.2%) out of 9 patients with microscopic or no tumor. The median DFS was 11.0 months with macroscopic tumor and 16.0 months with microscopic or no tumor (log rank test *p* = 0.029) (Fig. 3).

**Fig. 4:**
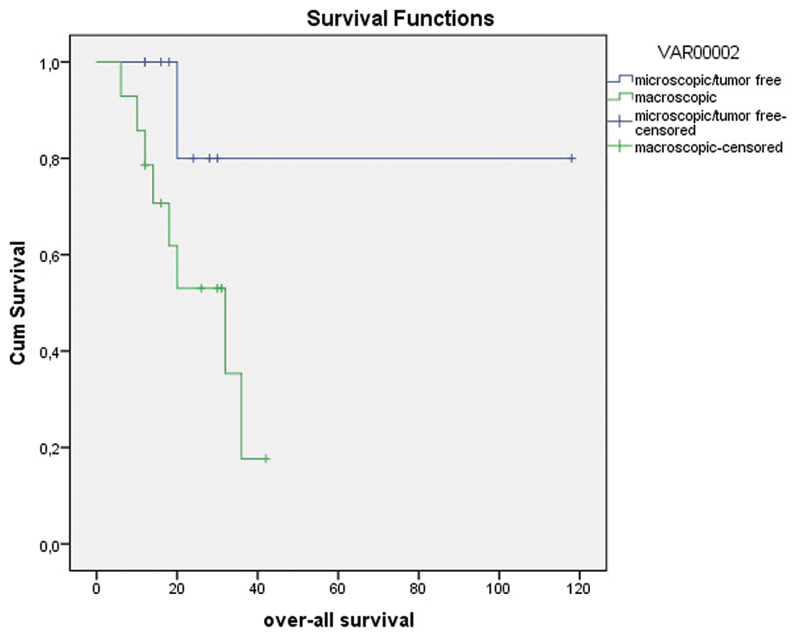
Overall survival graph with regard to presence of macroscopic tumor in the pathologic specimen (months).

The number of patients was not adequate to calculate the median overall survival duration by Long Rank test analysis with regard to the presence of macroscopic tumor (Fig. 4).

## Discussion

Cotreatment with radiotherapy and surgery as primary approach is avoided in patients with cervical cancer in order not to increase morbidity. However, studies published in recent years have suggested that surgical approach following radiotherapy was acceptable and had no negative impact on morbidity and survival.^13^ ^15^ ^16^ ^17^ ^18^ These studies suggested surgical approach either for the purpose of completion of hysterectomy, when brachytherapy could not be performed optimally, or as an alternative to brachytherapy.

In the present study, we analyzed the findings in LACC patients who had undergone surgery due to persistence after CRT. Out of 23 cases assessed in the present study, 14 (60.8%) had relapsed. The median DFS duration was 15.0 ± 4.4 (95% CI: 6.3–23.6) months. All of the recurrences had happened within the first 2 years.

Size of tumor in the pathology specimen, histological subtype of tumor, surgical margin, operation type, whether brachytherapy had been administered or not, and occurrence of grade 3 complication were analyzed in terms of relation to recurrence (Table 4). Among these, surgical margins positivity was related to local recurrence only and the presence of macroscopic tumor in the pathology specimen to overall recurrence (*p* = 0.048 and *p* = 0.029, respectively). In the presence of gross tumor, distant recurrences could not be prevented even if negative surgical margins were reached. At this point, adjuvant systemic chemotherapy can be considered to be useful at preventing distant recurrences. When we analyzed for local recurrence, local control could be achieved even in the presence of gross tumor if surgical margin negativity could be achieved.

### Simple Hysterectomy? Radical Surgery?

In patients with central residual tumor detected in the evaluations after CRT, there is no certain view about the preference between simple hysterectomy and more radical operation as the required surgical treatment. In the present study, there was no statistical difference between patients who had undergone simple hysterectomy or more radical surgery, in terms of both distant and local recurrences (Tables 5 and 6). According to this result, it might be important to avoid radical surgery not to increase morbidity unless a survival benefit is obtained. However, when simple hysterectomy is performed to avoid the morbidity of radical surgery, the risk of surgical margin positivity, which is a negative determinant of survival, might be augmented. In the present study, surgical margin positivity was found to be 70% (7 out of 10 patients) in radical surgery and 50% (3 out of 6 patients) in simple hysterectomy. Statistical analysis was not performed due to the limited number of patients. We observed in the present study that surgical margin negativity was quite significant for survival. The median DFS was estimated to be 7.0 months with positive surgical margins and 24.0 months with negative surgical margins (log rank test *p* = 0.048) (Fig. 1). Accordingly, it can be suggested that it is best to perform surgery as extended as possible to attain negative surgical margins and to improve survival. Attaining surgical margin negativity is more likely with extended surgery. On the other hand, detecting surgical margin positivity intraoperatively is not possible, especially in irradiated tissues. In a study by Boers et al,^19^ surgical margin negativity could be achieved in 53 out of 61 patients in spite of performing radical surgery. In this respect, frozen section evaluation may be guiding; however, it is not practical and can be misleading.

Our purpose in surgical treatment should be to achieve negative surgical margins; however, ensuring it intraoperatively is difficult in this patient population. The surgeon may perform simple hysterectomy if surgical margin negativity can be ensured. Boers et al^19^ reported that radical surgery did not improve survival in patients with central residual tumor and they did not recommend radical surgery for these patients. To sum up, we believe that the type of surgery should be decided according to the intraoperative evaluation. Moreover, initial stage of disease, imaging findings after CRT, and clinical examination findings should be definitely considered. In the present study, attaining negative surgical margins, rather than the operation type, appeared to be important. Surgical marginal positivity that may be encountered while avoiding radical surgery may result in poor prognosis.

### Complications

In the present study, this surgical treatment following CRT was not quite pleasing in terms of postoperative complications. Several studies reported acceptable levels of postoperative complication rate, but in the present study, grade 3 complications were observed in 52% of the patients.^16^ By examining further, we analyzed the period between the last session of radiotherapy and the surgery administered to the patients. Grade 3 complications had occurred in 3 (33.3%) out of 9 patients when operated within the first 2 months and in 9 (64.3%) out of 14 patients when operated after 2 months. Due to the small number of patients, statistical analysis could not be done; however, the complication rate had increased almost twofold in patients operated after the first 2 months. This might be attributed to the timing of surgery after the development of radiation fibrosis. Fibrosis, defined as the overaccumulation of collagen in tissues due to radiation injury, is a late-term complication of radiotherapy.^20^ Complications include difficulty of bleeding control in fibrotic tissue, injury to neighboring organs due to inability to discern tissue planes, delay in wound healing, and increase of fistula development. Some publications suggested that pentoxifylline or vitamin E were beneficial in the treatment of fibrosis.^21^ ^22^ ^23^ ^24^

Depending on the improvements in radiotherapy techniques, a better tissue-radiation dose relationship may result in less tissue damage, especially in disease-free tissues. This may be an explanation for studies in which surgical approach following radiotherapy was acceptable and had no negative impact on morbidity. The frequency of grade 3 complications was not low in our study; however, these complications had no effect on survival. Even though survival was not affected, performing surgery as soon as possible (i.e., before the development of radiation fibrosis) when planned in these patients might be important to prevent complications. In the present study, complication rates were similar in patients with simple hysterectomy and in those with a radical surgery. Grade 3 complications had occurred in 10 out of 17 patients with simple hysterectomy and in 2 out of 4 patients with radical surgery. We believe that a statistical difference could not be demonstrated due to the limited number of our patients. A generally accepted view is that complications increase as surgery is extended. In 34 patients who underwent radical hysterectomy after primary radiotherapy (15 patients for persistent and 19 patients for recurrent disease), Maneo et al^25^ estimated the rate of grades 3 and 4 complications to be 44%, the 5-year OS rate to be 49%, and the median survival to be 22 months; they suggested radical surgery as an alternative procedure to exenteration in selected patients.

In the present study, the presence of macroscopic tumor in the pathology specimen was found to be another determinant of recurrence. When evaluating the response to treatment after CRT, residual tumor volume was considered. A large volume of residual tumor is an indicator of poor response and negatively influences survival. In the present study, the median DFS was found to be 11.0 months in the presence of gross tumor after CRT and 16.0 months with microscopic/no tumor (Log-Rank test *p* = 0.029) (Fig. 3). The present study shows that, if surgical margin negativity was attained, the presence of gross tumor was not a risk factor for local recurrence; however, it seemed to be a risk factor for distant recurrences. In our opinion, distant recurrences were not associated with surgery treatment. We think that local recurrences could be linked to surgical treatment. Postoperative systemic chemotherapy to prevent distant recurrences might be argued for. There has been no study on this issue.

In the present study, the pathologic examination had reported no tumor in 21.7% of the patients, tumor cells at microscopic scale in 17.3%, and macroscopic tumor in 60.8%. In spite of being operated due to residual tumor, the pathologic examination had revealed no tumor in 21.7% of the patients. In a similar study, tumor could not be demonstrated in 28% of hysterectomy materials even though preoperative biopsy had revealed it.^19^ This fact could be attributed to the fallibility of diagnostic techniques but also to the continuation of tumor regression even until the day of surgery because of the long-term ongoing therapeutic effect of radiotherapy. Indeed, the early phase after radiotherapy is a period in which diagnostic methods including both biopsy and imaging might be misleading, making an accurate diagnosis troublesome. Tumor regression in the course of time is possible. However, during this waiting time, treatment may be delayed in the presence of a real residual tumor, radiation fibrosis may develop, and postoperative complications may increase. We believe that studies about the best method to make the diagnosis most accurately in this period are needed.

Brachytherapy, which is part of CRT, might not be administered for various reasons. Some studies pointed out that hysterectomy could be performed as a completion surgery in this situation.^16^ ^17^ ^18^ In our study, 9 patients could not receive brachytherapy (residual tumor in 7 and closed cervical canal in 2). Out of these patients, 8 had received external boost therapy. In our patient group, whether brachytherapy had been administered or not was found to be insignificant with regard to recurrence (Tables 4 and 5).

The main limitation of the present study included the absence of a comparative group (i.e., patients with a suspicious residual tumor who did not undergo hysterectomy). However, this problem may be overcome by comparing two groups with randomized prospective studies, but such a study is currently absent.

## Conclusion

To conclude, clinical experience in the population of LACC patients with CRT-resistance is not sufficient and there is no recommended standard treatment. We think that the treatment should be personalized. Simple hysterectomy and radical hysterectomy are options. However, grades 3 and 4 complication rate of performed surgery is high. Diagnostic techniques (imaging or biopsy) may be more misleading in the period following radiotherapy; therefore, the selection of patients who will be referred to surgery is not clear. The presence of macroscopic tumor in the pathology specimen and positive surgical margins are poor prognostic factors. The most important determinant of survival is to achieve negative surgical margins rather than radical surgery or simple hysterectomy.
